# Supplementary material for: Fluorescent sp3 Defect-Tailored Carbon Nanotubes Enable NIR-II Single Particle Imaging in Live Brain Slices at Ultra-Low Excitation Doses
Source: Sci Rep. 2020 Mar 24;10:5286. doi: 10.1038/s41598-020-62201-w (PMC7093457; doi:10.1038/s41598-020-62201-w)
Supplement: Supplementary file 1 — Supplementary Dataset 1. [file 41598_2020_62201_MOESM1_ESM.docx]

**SUPPLEMENTARY INFORMATION**

**Fluorescent sp^3^ Defect-Tailored Carbon Nanotubes Enable NIR-II Single Particle Imaging in Live Brain Slices at Ultra-Low Excitation Doses**

Amit Kumar Mandal^1,2^, Xiaojian Wu^3^, Joana S. Ferreira^4,5^, Mijin Kim^3^, Lyndsey R. Powell^3^, Hyejin Kwon^3^, Laurent Groc^4,5^, YuHuang Wang^3^, Laurent Cognet^1,2,*^

*^1^Université de Bordeaux, Laboratoire Photonique Numérique et Nanosciences, UMR 5298, 33400 Talence, France.*

*^2^Institut d’Optique & CNRS, LP2N UMR 5298, 33400 Talence, France.*

*^3^Department of Chemistry and Biochemistry, University of Maryland, College Park, MD 20742, United States*

*^4^Université de Bordeaux, Interdisciplinary Institute for Neurosciences, UMR 5297, 33076 Bordeaux, France.*

*^5^CNRS, IINS UMR 5297, 33076 Bordeaux, France.*

***Correspondence** should be addressed to: Laurent Cognet. Email: laurent.cognet@u-bordeaux.fr

**Content:**

- **Supplementary Figure 1**
- **Supplementary Figure 2**

**
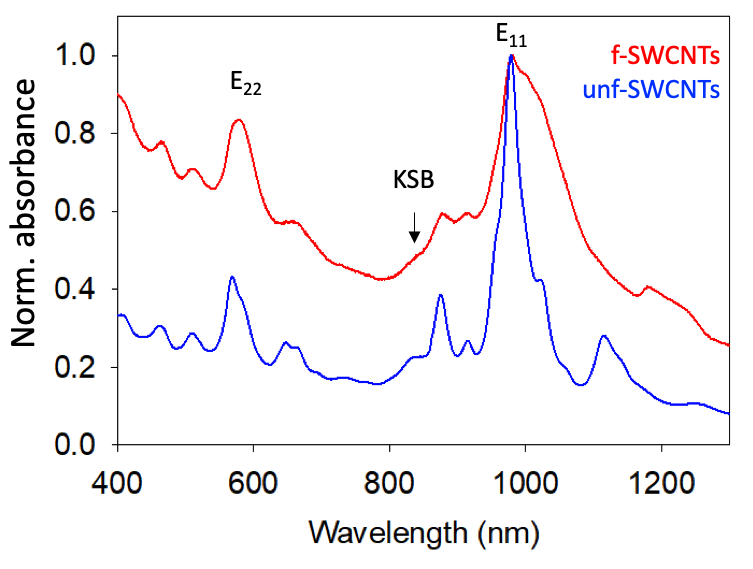
**

**Supplementary Figure 1:** Absorption spectrum of the nanotubes used in this study (CoMoCAT SG65i) suspended in DOC. E_11_, KSB and E_22_ bands corresponding to (6,5) SWCNTs, the most abundant chirality in these samples, are indicated. As previously observed, functionalization induces decreased and broadened absorption peaks.^1^


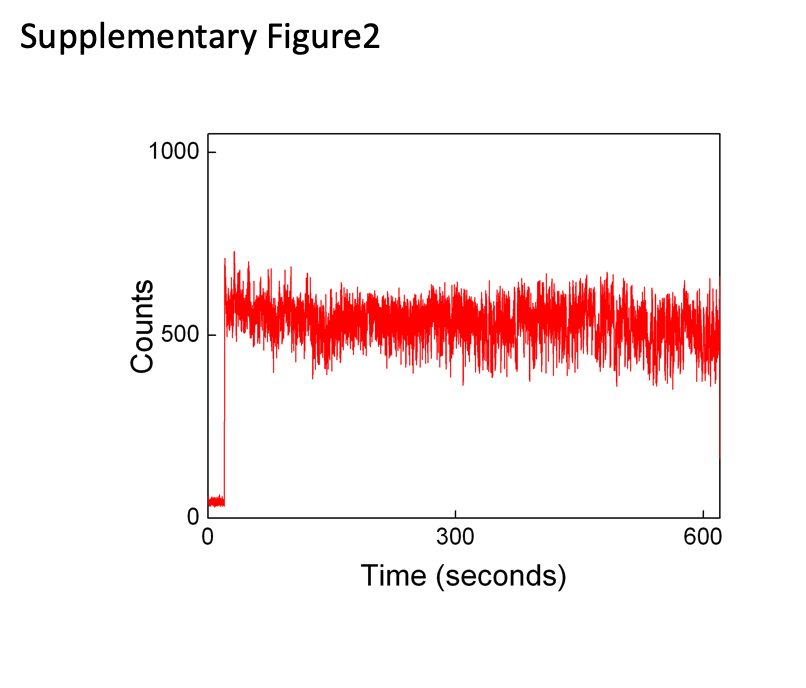
 **Supplementary Figure 2:** Luminescence photostability of individual f-SWCNTs for up to 10 minutes under continuous excitation at 985 nm. At ~ 20 seconds, the excitation light is applied.

**References:**

1. Piao, Y., Meany, B., Powell, L. R., Valley, N., Kwon, H., Schatz, G. C., Wang, Y. Brightening of carbon nanotube photoluminescence through the incorporation of sp^3^ defects. *Nat. Chem.* **5**, 840-845, (2013).
